# Autonomic Reflexes With Epipharyngeal Abrasive Therapy (EAT)

**DOI:** 10.7759/cureus.77575

**Published:** 2025-01-17

**Authors:** Ito Hirobumi

**Affiliations:** 1 Otolaryngology, Ito ENT Clinic, Funabashi, JPN

**Keywords:** autonomic nerve activity regulation, baroreceptor reflex, chronic epipharyngitis(nasopharyngitis), eat reflex, epipharyngeal abrasive therapy (eat), parasympathetic nerve stimulation, pharyngeal reflex, sympathetic nerve stimulation, trigeminal autonomic reflex, vagal cardiac reflex

## Abstract

A literature review was conducted of epipharyngeal abrasive therapy (EAT) in the treatment of chronic epipharyngitis, focusing on the mechanism of action by autonomic nerve stimulation. The mechanism of action of EAT in stimulating the immune system has recently become clear. However, the mechanism of action of EAT on the autonomic nervous system remains to be elucidated. The purpose of this paper is to provide insight into the still not fully understood mechanism of EAT stimulation of the autonomic nervous system in chronic epipharyngitis by collecting and integrating previous studies and papers focusing on the autonomic nerve stimulating effects of EAT. The EAT stimulates a network of brainstem neurons involved in swallowing, vomiting, breathing, and circulatory centers, and further affects endocrine reflexes via the hypothalamus and pituitary, and stress responses via the limbic system.

The EAT reflex is hierarchically integrated and is thought to reflexively control not only the pharyngeal reflex but also autonomic functions such as airway, breathing, cardiovascular, cerebral circulation, digestive, and endocrine glands. The immune system, endocrine system, and autonomic nervous system are thought to interact with each other to produce the therapeutic effect of EAT. It is important to determine which mechanism is predominantly involved in each case of chronic epipharyngitis and to utilize it in treatment. Elucidating the mechanism of action of EAT autonomic nerve stimulation will be an important guideline in determining the treatment strategy for chronic epipharyngitis.

## Introduction and background

Epipharyngeal abrasive therapy (EAT) [1] is a treatment for chronic epipharyngitis (also known as nasopharyngitis) [2]. Since EAT is effective in improving a variety of autonomic symptoms [3], it is possible that its therapeutic effects are expressed via autonomic reflexes. However, there are few reports on the mechanism of action of EAT by the autonomic nervous system stimulation [4-7]. Autonomic function is often controlled reflexively via the central nervous system. Reflex pathways are classified into four categories depending on whether the afferent or efferent pathways pass through the autonomic or somatic nervous system: somatic-visceral reflexes, visceral-visceral reflexes, visceral-somatic reflexes, and somatic-somatic reflexes. Reflexes triggered by mechanical stimulation of the oral and nasal cavities occur in the pharyngeal reflex, swallowing reflex, salivary reflex, baroreceptor reflex, respiratory reflex by stretch receptors in the lungs and chemoreceptors in the blood vessels, sneeze reflex, cough reflex, and pain reflex by nociceptive stimulation. Afferent inputs interfere within the central nervous system and are integrated into efferent outputs. Reflex pathways exist in the autonomic and somatic nervous systems, although some reflexes are independent of each system, and some reflexes straddle both systems. In addition, reflex modulation often occurs with the integration of several reflexes rather than just one. In addition, reflexes that regulate hormone secretion may be elicited [8,9], and the EAT reflex is thought to elicit and integrate a variety of autonomic reflexes, including the visceral-visceral and visceral-somatic reflexes listed above. This paper collects, integrates, and discusses past studies and papers focusing on autonomic reflexes induced by EAT. The purpose was to provide insight into the relationship between EAT reflexes and EAT treatment effects, which are not yet fully understood.

## Review

Pharyngeal reflex induced by EAT

EAT is performed by nasal or oral abrasion, and stimulation of the epipharyngeal mucosa by EAT stimulates the autonomic nervous system to induce the pharyngeal reflex. Afferent stimulation by nasal abrasion induces a pharyngeal reflex, which is a reflex that is triggered by the cribriform nerve (first branch of the trigeminal nerve), the posterior nasal nerve (second branch of the trigeminal nerve), the nucleus tractus solitarius (NTS), the spinal trigeminal nucleus, superior salivary nucleus (SSN), and locus coeruleus (LC). Afferent stimulation by oral abrasion provides input via the pharyngeal plexus (glossopharyngeal, vagus, and cervical sympathetic nerves) to the nucleus of the NTS tract and the trigeminospinal tract. These inputs are transmitted to the nucleus of suspicion and output via the pharyngeal plexus to the vagus motor branch and the glossopharyngeal nerve motor branch to induce reflex muscle contractions, resulting in the pharyngeal reflex. The pharyngeal reflex by EAT is considered a visceral-somatic reflex [10]. The pharyngeal reflex, together with other airway-gastrointestinal reflexes such as the swallowing reflex, is considered a protective reflex that helps prevent airway obstruction by preventing oral material from entering the pharynx [8].

Swallowing is a series of movements to transport the food mass from the oral cavity to the stomach. The pharyngeal phase, which is carried out between 0.6 and 0.8 seconds, is a reflex movement, and the pharyngeal reflex forms part of the swallowing movement. During this period, a series of reflexes such as nasopharyngeal space closure by soft palate elevation, laryngeal elevation, glottal closure, driving of the food mass by pharyngeal contraction, and opening of the esophageal inlet by relaxation of the cricopharyngeal muscle, are controlled by the central pattern generator (CPG) for swallowing in the medulla oblongata [8]. Nasopharyngeal closure is accomplished by posterior upward movement of the soft palate and involves the coordinated involvement of the palatine sail elevator muscle, palatoglossus muscle, pterygoid muscle, and palatopharyngeal muscle, all of which are innervated by the pharyngeal plexus. EAT may strengthen the pharyngeal reflex, thereby enhancing nasopharyngeal closure function and possibly helping to prevent aspiration. During swallowing, the glottis is reflexively closed and the lower airway is protected from aspiration. Breathing, speech, and swallowing are closely interrelated through interregulatory mechanisms. EAT is thought to influence swallowing, breathing, and speech functions. The mandibular nerve, the third branch of the trigeminal nerve, is involved in masticatory movements, and the tensor veli palatini muscle is innervated by the mandibular nerve. The tensor veli palatini muscle attaches to the palatoglossus tendonulus, and when a food mass compresses the palatoglossus tendonulus, the palatoglossus tendonulus is stretched, causing a reflex contraction of the tensor veli palatini muscle. During swallowing movements and yawning, the tensor veli palatini muscle contracts to open the Eustachian tube pharyngeal opening, and the Eustachian tube is released [8]. Swallowing movements promote Eustachian tube opening and also affect middle ear function. EAT is thought to influence Eustachian tube and middle ear function as well [8].

EAT stimulation also projects directly and indirectly to the midbrain, hypothalamus, amygdala, hippocampus, and frontal lobes. EAT stimulates neuronal networks in the brainstem that constitute the swallowing, vomiting, respiratory, and circulatory centers, and further affects endocrine reflexes via the hypothalamus and pituitary gland and stress responses via the limbic system. The EAT reflex is hierarchically integrated and is thought to reflexively control not only the pharyngeal reflex but also autonomic functions such as airway, breathing, cardiovascular, cerebral circulation, digestive, and endocrine glands [11]. In addition, since the nasopharynx, together with the palatine tonsils, constitutes Waldeyer's tonsil ring, chronic epipharyngitis may involve the same immunologic mechanisms as tonsillar focal disease. The effects of EAT on the immune system have also been reported [12-14], and it is thought that EAT stimulation affects the immune, endocrine, and autonomic nervous systems, interacting with each other to produce a therapeutic effect on chronic epipharyngitis [15,16].

Regulation of cerebral circulation

The brain requires large amounts of oxygen and energy, but its energy stores are low, so it needs to obtain energy mainly from glucose in the blood. Therefore, cerebral blood flow is maintained at a constant level by cerebral autoregulation. Within the blood pressure range of 60-150 mmHg, blood vessels contract and relax in a blood pressure-dependent manner. The concept of cerebral circulatory regulation function, in which cerebral blood flow homeostasis is maintained, was reported by Lassen et al [17].

Metabolic regulation of cerebral circulation is mediated by carbon dioxide and other substances resulting from brain cell activity. Cerebral blood vessels are sensitive to the partial pressure of oxygen and carbon dioxide in the blood. Blood gas status is sensed by two peripheral chemoreceptors in the carotid and aortic bodies, which are O2 sensors, and the central chemosensitive area, which is a CO2 partial pressure sensor in the ventral lateral area of the medulla oblongata. These are sensed by respiratory networks in the brainstem. This information is transmitted to the respiratory network in the brainstem, which provides feedback control to optimize respiratory output [18]. It has also been reported that astrocytes act as sensor cells that sense hypoxia and play an important role in maintaining brain function and respiratory output [19,20].

The rostral medullary ventral lateral area contains respiratory and circulatory centers. The respiratory rhythm is reflected in the spontaneous activity of sympathetic nerves distributed throughout the body, and as a result, respiratory variations are observed in blood pressure. Heart rate also shows respiratory variations in sync with respiration. Heart rate decreases during expiration and increases during inspiration. It is thought that the parasympathetic nervous function increases during expiration and the sympathetic nervous function increases during inspiration in synchronization with respiration. Respiratory and cerebral circulatory functions influence each other [21,22]. It is possible that the autonomic stimulation effect may differ depending on whether EAT stimulation is performed during the expiratory or inspiratory phase and which region is stimulated.

Cerebral circulation is also regulated by the neurogenic regulation of autonomic nervous activity. The nerve fibers that regulate the cerebral circulatory system are broadly classified into intrinsic innervation, which originates in the brain itself, and extrinsic innervation, which originates in ganglia outside the brain. Intrinsic innervation and extrinsic innervation interact to neurally regulate vasoreactivity [23].

The nucleus basalis of Meynert (NBM) in the basal forebrain is innervated by nerve fibers that use acetylcholine (Ach) as a neurotransmitter. Cholinergic nerves from the nucleus Meynert act as vasodilators to regulate regional blood flow in the cortex. Somatosensory stimulation of the skin in various areas, as well as stimulation of the forelimbs and hindlimbs, increases cerebral blood flow. Regulation of regional blood flow is important for the development of higher cerebral functions. Increased cerebral blood flow is thought to be mediated by cholinergic innervation of the Meynert nucleus. The LC is innervated by nerve fibers that use noradrenaline (NA) as a neurotransmitter. The raphe nucleus is innervated by serotonin (5-hydroxytryptamine: 5-HT) neurotransmitter fibers. Noradrenergic nerves originating in the LC and serotonergic nerves originating in the nucleus coeruleus act in a vasoconstrictive manner. Astrocytes also connect the vasculature to neurons and provide neurogenic regulation of the cerebral circulatory system [24].

As extrinsic innervation, sympathetic nerve fibers that exert contractile effects on cerebral vessels originate in the superior carotid ganglion (SCG) and ascend along the internal carotid artery to become the internal carotid artery nerve, which is distributed to the cerebral vessels. This nerve is noradrenergic and acts vasoconstrictively via alpha receptors. Stimulation of cervical sympathetic nerves increases the extent of autoregulation that keeps cerebral blood flow stable in response to blood pressure fluctuations, suggesting that sympathetic activity has a protective role for the brain during rapid hypertension [25].

Parasympathetic nerve fibers are distributed throughout the cerebral vasculature, originating primarily from the pterygopalatine ganglion (SPG), the otic ganglion (OG), and the internal carotid plexus. These nerves are cholinergic and act in a vasodilatory manner. Vasoactive intestinal peptide (VIP) and nitric oxide (NO) also coexist and act as vasodilators. Stimulation of parasympathetic postganglionic fibers derived from the SPG increases blood flow in the cerebral cortex [26].

The afferent fibers of cerebral blood vessels enter the trigeminal ganglion (TG), internal carotid plexus, and dorsal root ganglia. Retrograde stimulation of trigeminal afferent fibers activates trigeminal nerve endings around cerebral blood vessels, releases calcitonin gene-related protein (CGRP) and other proteins, dilates cerebral blood vessels, and induces neurogenic inflammation [27]. Irritation of the epipharyngeal mucosa due to chronic epipharyngitis may be one of the causes of trigeminal neuralgia and headache.

EAT affects sympathetic and parasympathetic nerves via inputs from visceral afferent fibers. EAT also affects noradrenergic innervation [10]. EAT is thought to affect cerebral circulation by acting on both intrinsic and extrinsic innervation [23]. The regulatory effects of cerebral circulation on autonomic nervous activity are shown in Table 1.

**Table 1:** Regulation of cerebral circulation by autonomic nervous activity Cerebral circulation is neurogenically regulated by intrinsic innervation, which originates in the brain itself, and extrinsic innervation, which originates in ganglia outside the brain. EAT and INSPIGS work to regulate cerebral circulation by influencing autonomic nervous system activity. SPG/OG: sphenopalatine ganglion/otic ganglion; TG: trigeminal ganglion; SCG: superior cervical ganglion; INSPIGS: intranasal sphenopalatine ganglion stimulation; EAT: epipharyngeal abrasive therapy

Cerebral Circulatory Regulation	
Intrinsic Innervation	Nucleus Basalis of Meynert
	Locus Coeruleus
	Raphe Nuclei
	Astrocyte
	Local Interneuron
Extrinsic Innervation	SPG/OG
	TG
	SCG
Neuromodulation	INSPIGS
	EAT

About EAT and INSPIGS

The SPG is one of the important extracranial parasympathetic ganglia and is located in the pterygopalatine fossa, hanging over the maxillary nerve in the pterygopalatine fossa canalis. The SPG contains parasympathetic and sympathetic fibers and visceral afferent fibers. Parasympathetic fibers with cell bodies in the supra-salivary nucleus pass through the greater pyramidal nerve and pterygoid nerve and are replaced by postnodal fibers in the SPG, which are distributed in the nasal cavity as the posterior nasal nerve. Postnodal fibers from the SPG enter the cranial cavity through the ethmoid foramen and innervate cerebral blood vessels. The anterior, middle, and posterior cerebral arteries and basilar artery are regulated by NO-activating neurons from the SPG. Sympathetic fibers with cell bodies in the lateral horns of the thoracic medulla alternate with postnodal fibers in the superior cervical ganglion and are distributed as postnasal nerves via the deep pyramidal nerve, pterygoid nerve, and SPG. Some of the visceral afferent fibers of the trigeminal, vagus, and glossopharyngeal nerves provide afferent input to the center via the SPG. The SPG is thought to play an important role in cerebral circulation regulation [28].

Cluster headaches and related disorders are thought to involve the trigeminovascular system, trigemino-autonomic reflexes, and the hypothalamus, and are classified as trigeminal and autonomic headaches (also known as trigeminal automatic cephalalgias (TACs)) [29]. One of the possible causes is trigeminal-parasympathetic hyperreflexia, in which the intracranial parasympathetic nervous system is overexcited via SPGs [30]. SPG-block has been used as a treatment for this problem; Schoenen et al. developed SPG electrostimulation (SPG-Stim), which suppresses parasympathetic nerve activity by depleting neurotransmitters through high-frequency electrical stimulation of SPGs. SPG-Stim is thought to improve TACs by deactivating the interaction between the parasympathetic and trigeminal nervous systems through modulation of parasympathetic neurotransmission [31].

Jürgensh and May reported that SPG high-frequency stimulation may have an immediate effect during the acute phase of TACs, additionally, they found that repeated stimulation may induce neuroplastic changes and develop long-term modulatory effects on the parasympathetic nervous system [32]. Cheyuo et al. reported neuroprotective effects of vagus nerve stimulation (VNS), cholinergic anti-inflammatory pathways, and activation of cerebral vascular tone regulation as effects of parasympathetic nervous system activation [33]. Activation of the parasympathetic nervous system may antagonize various pathological mechanisms such as stroke.

EAT has been reported to have an effect on headaches [3], and since EAT stimulates both sympathetic and parasympathetic nerves, as well as trigeminal afferent fibers [10], it may stop the interaction between the parasympathetic and trigeminal nervous systems, suppressing excessive trigeminal-parasympathetic reflexes and improving headaches [31,32]. Activation of parasympathetic activity also relaxes cerebral vascular tone, improves cerebral circulation, and exerts an anti-inflammatory effect [33], suggesting that EAT may have a therapeutic effect on headaches by suppressing trigeminocardiac and autonomic reflexes.

Bahr-Hosseini and Saver reported the effects of SPG Stim on acute ischemic stroke. They reported the following effects of SPG Stim on acute ischemic stroke: (1) collateral vasodilation and enhancement of cerebral blood flow by the release of the vasodilating neurotransmitters NO and ACh, (2) reduction of edema by stabilization of the blood-brain barrier dependent on stimulation frequency and intensity, (3) anti-inflammatory, antiapoptotic and anti-excitatory effects by direct acute neuroprotection against the activation of the central cholinergic system, and potentiation of central cholinergic and adrenergic neuromodulation in cortical networks. excitatory effects, (4) potentiation of central cholinergic and adrenergic neuromodulation of cortical networks, and potentiation of neuroplasticity by NO release to stimulate neurogenesis [34].

Tanaka proposed intranasal sphenopalatine ganglion stimulation (INSPGS), which stimulates SPG via the endonasal route. The author reported that INSPIGS acts similarly to SPG electrical stimulation therapy, and that mechanical SPG stimulation with INSPGS may physiologically block the parasympathetic efferent pathway and block the efferent pathway of trigeminal-autonomic reflexes [35,36]. In other words, INSPIGS may exert its effects by suppressing trigeminal-parasympathetic reflexes by stopping the interaction between the parasympathetic and trigeminal nervous systems through the regulation of parasympathetic transmission.

Abrasive stimulation of the canopy and lateral wall of the nasopharynx by EAT is thought to stimulate visceral afferent fibers via SPGs innervating the nasopharyngeal mucosa, thereby affecting sympathetic and parasympathetic nerve activity [10]. Sympathetic stimulation by EAT acts vasoconstrictively [4], whereas parasympathetic stimulation acts vasodilatory by expressing the coexistent transmitter NO [8]. Parasympathetic stimulation also acts on sympathetic nerves to inhibit NA release and suppress vasoconstriction [8]. EAT also acts as a vasodilator by retrogradely stimulating trigeminal afferent fibers that innervate cerebral blood vessels [8]. As a result, EAT may improve cerebral blood flow. EAT may also stimulate SPGs and inhibit trigeminal-parasympathetic reflexes similar to INSPIGS, thereby improving symptoms such as vasogenic headache [35,36].

Furthermore, EAT may stimulate the solitary bundle nucleus by stimulating vagal visceral afferent fibers, thereby exerting VNS-like effects. EAT may stimulate glial cells that control the brain environment, creating neuroplasticity in the brain and promoting learning and rehabilitation [37,38]. The effects of EAT on autonomic symptoms may be due to its VNS-like effects, along with its ability to regulate cerebral circulation and improve cerebral blood flow.

Autonomic regulation of the cardiocirculatory system

The cell bodies of sympathetic preganglionic neurons are located in the intermediolateral cell column (IML) of the spinal gray matter from the first thoracic medulla to the second lumbar medulla. Myelinated preganglionic fibers from this cell body pass through the anterior root of the spinal nerve and then through the white traffic branch to the paravertebral ganglia (trunk ganglia) in the sympathetic trunk. Neurons are replaced in the superior cervical ganglion, middle cervical ganglion, and stellate ganglion to innervate the heart and blood vessels (mainly muscular arteries, small and small arteries, and veins). There is no innervation of capillaries. Sympathetic impulses to blood vessels are continuous, and the amount of impulse changes the degree of vascular tone. Sympathetic stimulation causes peripheral vascular resistance to increase as small arteries and microvessels contract. As a result, blood pressure increases. Sympathetic nerves also innervate the veins, and when the veins contract with sympathetic stimulation, venous volume decreases, blood in the veins as a volume reservoir moves to the right atrium (increasing venous return), and cardiac output increases. Sympathetic stimulation of the heart acts as a positive inotropic effect by increasing heart rate, increasing cardiac contractility, and shortening the conduction time [8].

The cell bodies of parasympathetic preganglionic neurons are located in the brainstem and sacral spinal cord. Pre-nodal fibers exit the brainstem with the third, seventh, ninth, and tenth (vagus) cranial nerves and exit the spinal cord at the S2 and S3 levels. Parasympathetic ganglia (ciliary, pterygopalatine, otolaryngeal, pelvic, and vagus ganglia) reside within the effector apparatus, and postnodal fibers are short. Thus, the parasympathetic nervous system can elicit specific and localized responses to the effector. The heart is innervated by the vagus nerve. Vagus nerve stimulation decreases heart rate, cardiac contractility, and excitation conduction velocity. These are referred to as negative afferent effects. Sympathetic nerves innervate the atria and ventricles, whereas vagus nerves innervate mainly the sinoatrial node, atrial muscles, and atrioventricular node, with less innervation of ventricular muscles. Parasympathetic nerves are also distributed in the coronary arteries and cause endothelium-dependent vasodilation by releasing NO from endothelial cells. Parasympathetic nerves also innervate the sacralis and innervate the circulatory system by dilating vessels in the pubic region [9]. 

EAT has both sympathetic and parasympathetic stimulating effects. However, the effects are antagonistic, and it is still unclear which stimulating effect is dominant. Differences in stimulus intensity, timing of stimulation, location of stimulation, and individual sensitivity may be involved.

There are short-term and long-term blood pressure regulation mechanisms in systemic blood pressure regulation. The short-term regulatory mechanism is a neural regulatory mechanism to restore normal blood pressure to its normal level in a short period of time after sudden blood pressure fluctuations caused by postural changes, exercise, straining, or mental agitation. The long-term regulatory mechanism is a humoral regulatory mechanism that regulates salt and water excretion from the kidneys. The baroreceptor reflex is a neural blood pressure regulatory mechanism that rapidly restores blood pressure fluctuations. The cardiopulmonary baroreflex, a low-pressure system, and the carotid sinus baroreflex and aortic baroreflex, which are high-pressure systems, are known [8].

The cardiopulmonary baroreflex system is a reflex system mediated by the baroreceptors in the pulmonary veins and right and left atria via the vagal afferent pathways. The pulmonary veins and right and left atria are low-pressure systems, and the excitation of cardiopulmonary baroreceptors with increased plasma volume reaches the hypothalamus via vagal afferent pathways and suppresses vasopressin secretion. Inhibition of vasopressin secretion inhibits water reabsorption, increases urine output, and reduces fluid volume, resulting in a hypotensive effect. A portion of the cardiac-pulmonary baroreceptor excitation is also input to the medullary solitary bundle nucleus via the vagal afferent tract and is involved in neurogenic blood pressure regulation, although its regulatory power is said to be weaker than that of the hyperbaric system [8].

The baroreceptor reflex is a negative feedback system that decreases blood pressure to its original value by decreasing heart rate and peripheral sympathetic nerve activity when blood pressure increases rapidly, and increases heart rate and sympathetic nerve activity to its original value when blood pressure decreases rapidly. A rapid rise in blood pressure stretches the arterial wall and excites baroreceptors in the carotid sinus and aortic arch, triggering the baroreflex. Action potentials generated in the carotid sinus baroreceptors are transmitted from the carotid sinus nerve via the glossopharyngeal nerve to the solitary bundle nucleus of the medulla oblongata. Action potentials in the aortic baroreceptors are similarly transmitted to NTS via the vagus afferent tract from the aortic nerve. This information is transmitted to the cardiovascular center in the medulla oblongata [9].

Input from NTS is transmitted to the caudal ventrolateral medulla (CVLM), and information from the CVLM is projected via inhibitory neurons to the rostral ventrolateral medulla (RVLM). The actions of these inhibitory neurons suppress the excitation of neurons in the RVLM and inhibit sympathetic activity. Inhibition of sympathetic activity decreases cardiac contractility and heart rate, dilates peripheral blood vessels, and lowers blood pressure. Conversely, during hypotension, baroreceptor excitation does not occur and the cardiovascular center is not inhibited, so the sympathetic nervous system is activated, blood pressure increases, heart rate, and cardiac contractility increase. The baroreceptor reflex acts as negative feedback on blood pressure fluctuations and controls them to be minimized [9].

The inhibitory neurons of CLMV are modulated by NO, which enhances inhibitory neuronal activity and suppresses sympathetic nervous system activity, resulting in hypotensive and heart rate-lowering effects. Reactive oxygen species (ROS), a molecular mechanism that antagonizes NO, attenuates inhibitory neuronal activity in CLMV and stimulates sympathetic nervous system activity. Elevated concentrations of angiotensin II in blood and cerebrospinal fluid are also detected in periventricular and subarachnoid organs, where the blood-brain barrier is relatively sparse. Thus, angiotensin II promotes ROS production and de-represses the RVLM by decreasing NO and activating the sympathetic nervous system. Since the balance between NO and ROS in the cardiovascular center plays a major role in sympathetic nervous system activity, the interaction between NO and ROS should be considered. The RVLM also receives inputs from higher centers such as the cerebral cortex and hypothalamus, which may ultimately determine sympathetic nervous system activity, including the effects of emotion and stress. It has been reported that reduced sensitivity of baroreceptor reflexes is associated with worse life expectancy in patients with heart failure [9].

The circulatory center regulates the contraction of the heart and blood vessels and controls blood pressure by regulating sympathetic and parasympathetic nervous activity. The heart and blood vessels are dually innervated by sympathetic and parasympathetic nerves, and their function is regulated by a combination of sympathetic and parasympathetic activities. Harada examined the immediate effects of EAT on vasomotor reflexes using the finger vasomotor reflex. First, EAT elicited a vasoconstrictor reflex due to increased sympathetic activity, and then a vasodilator reflex due to suppressed sympathetic activity. Cases of autonomic dysregulation such as orthostatic dysregulation and vertigo showed prolonged vasomotor reflex induced by EAT, and the authors reported improvement of autonomic symptoms and recovery of vasomotor reflex as a temporal effect of EAT [4]. Thus, EAT is thought to have both sympathomimetic and autonomic function-restoring effects.

**Figure 1:**
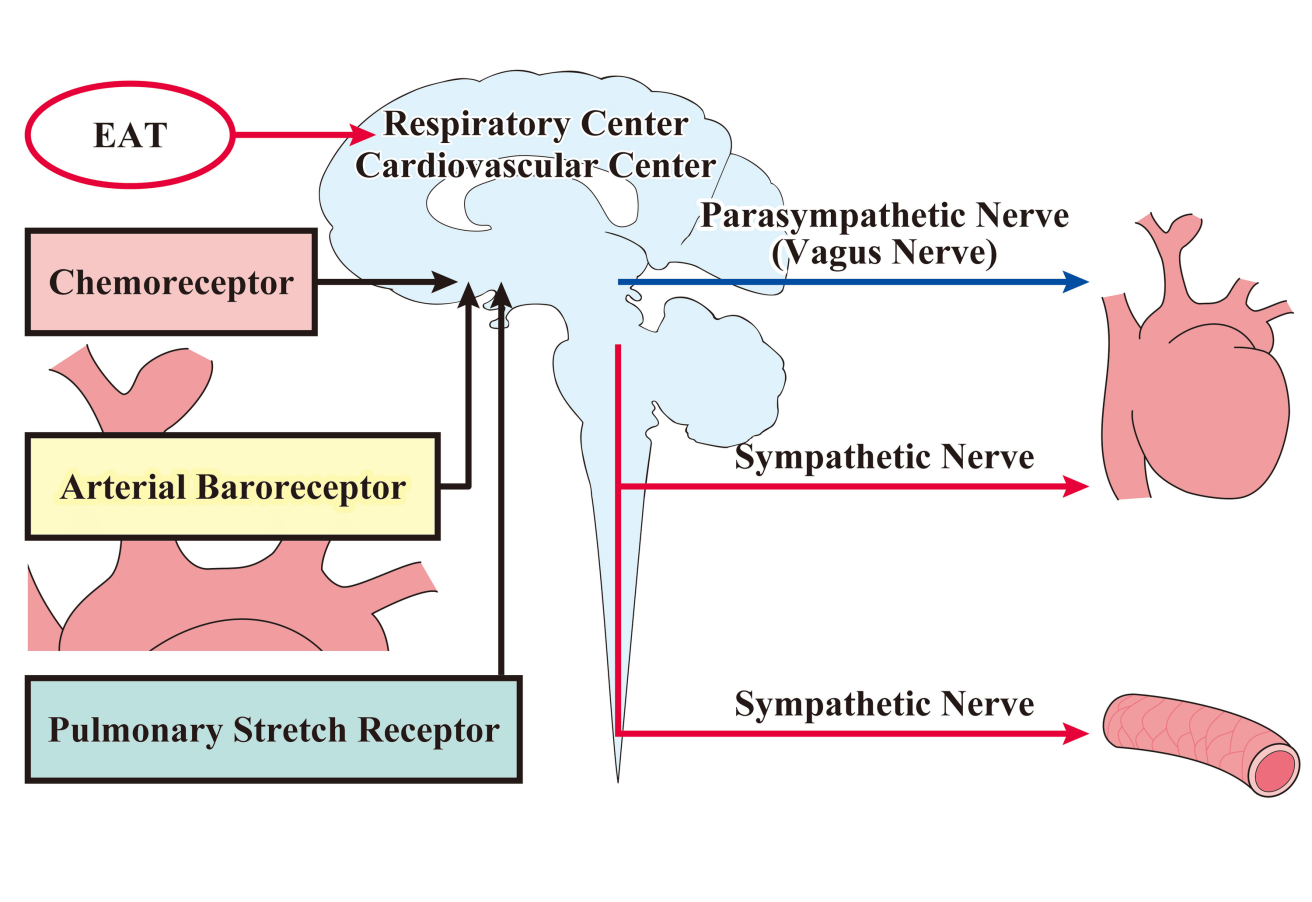
Regulation of Cardiovascular Reflexes by EAT In response to an increase in blood pressure, parasympathetic activity increases, cardiac activity is suppressed, and a bradycardia reflex is elicited. In response to a decrease in blood pressure, sympathetic activity increases, cardiac activity is enhanced, vasoconstriction occurs, and a tachycardia reflex is triggered. Afferent inputs from chemoreceptors and pulmonary stretch receptors are also added to regulate cardiovascular activity. EAT stimulation controls cardiovascular reflexes by influencing respiratory and cardiovascular centers. Image Credit: Author

Ito investigated autonomic reflexes induced by EAT using heart rate variability analysis and found that EAT has both sympathetic and parasympathetic stimulating effects as immediate effects, but that the response depends on the timing and site of stimulation. As a longer-term effect of EAT, the sum of autonomic activity is suppressed and parasympathetic activity is also suppressed [10]. Relatively, sympathetic activity is stimulated and sympathetic reflexes are reported to be more easily produced [6,7,10,15,16]. Parasympathetic effects on cardiac function are known to include a decrease in heart rate (negative alteration effect), myocardial contractility (negative alteration effect), and interventricular conduction velocity (negative alteration effect) [39]. EAT may increase cardiac function and facilitate the appearance of sympathetic reflexes by suppressing parasympathetic activity. Since the increase in cardiac function is thought to be important for life support, it is possible that the temporal effects of EAT stimulation may increase the reflex reactivity of cardiac function. The control of cardiovascular reflexes by EAT is shown in Figure 1.

Effects of EAT on blood pressure

EAT has the ability to stimulate both sympathetic and parasympathetic activity with immediate effect. As a time-dependent effect of counteracting stimulation, it inhibits overall autonomic activity and parasympathetic activity and promotes sympathetic activity [6,10]. Thus, the long-term effect of EAT is to depress overall autonomic activity, but sympathetic reflexes are activated more readily during stimulation. In addition, since the baroreceptor reflex is activated, blood pressure fluctuations are suppressed and blood pressure tends to decrease, suggesting that EAT may have a blood pressure regulating effect [6].

**Figure 2:**
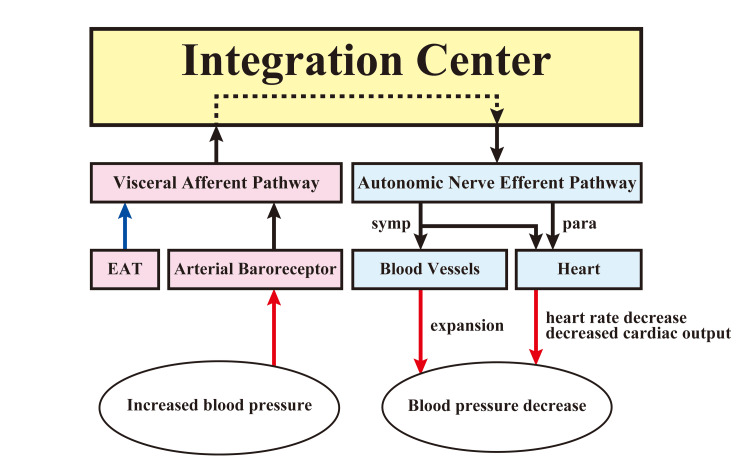
Effects of EAT-induced activation of the baroreceptor reflex on the cardiovascular system When blood pressure increases, the baroreceptor reflex is activated, triggering visceral- visceral reflexes to the blood vessels and heart. Sympathetic activity is inhibited and blood vessels dilate. Parasympathetic activity is stimulated, heart rate is reduced, and cardiac output is decreased. EAT stimulation is thought to suppress blood pressure fluctuations and maintain blood pressure homeostasis by activating the baroreceptor reflex. symp: sympathetic nerve system; para: parasympathetic nerve system

The baroreceptor reflex and sympathetic nervous system are important mechanisms for short-term blood pressure regulation, but they have also been reported to be involved in long-term blood pressure regulation. Dysregulation of the central sympathetic nervous system, including the baroreceptor reflex, is closely related to the development of hypertension, and high blood pressure variability itself is a risk factor for cardiovascular events. Baroreceptor reflex afferent pathway stimulation produces a response similar to that of high blood pressure input to the baroreceptors, causing the vasomotor center to decrease sympathetic nerve activity. Using this principle, a device has been developed to chronically activate the baroreceptor reflex by implanting a stimulating electrode on the adventitia of the carotid sinus [40]. The use of carotid sinus stimulators and renal sympathetic nerve ablation are effective for treatment-resistant hypertension [41]. Central circulatory regulation has been attempted by activating the baroreceptor reflex and regulating blood pressure through the intervention of the sympathetic nervous system [42]. In heart failure, the sympathetic nervous system is activated by peripheral and central mechanisms to form a vicious cycle, and appropriate treatment at the earliest possible stage is considered necessary. EAT may work to regulate blood pressure by intervening in sympathetic nervous system activity through the activation of vasomotor and baroreceptor reflexes. The activation of baroreceptor reflection by EAT is shown in Figure 2.

Heart rate variability represents the basal level of cardiac parasympathetic function and indicates static autonomic activity. Baroreceptor reflex sensitivity reflects cardiac sympathetic and parasympathetic reflex function and represents dynamic autonomic activity. EAT is thought to decrease basal parasympathetic activity but stimulate parasympathetic reflexes, which are dynamic autonomic activities antagonistic to sympathetic tone. Activation of parasympathetic function protects the myocardium and improves the prognosis of heart failure [43]; when baroreceptor reflexes are activated by EAT, sympathetic activity is suppressed and elevated pressure is reduced. As a result, EAT suppresses blood pressure variability, maintaining blood pressure and homeostasis. It is thought that EAT may suppress static autonomic activity but stimulate dynamic autonomic activity.

Effects of EAT on vestibular function

The central command (CC), which activates the somatomotor nervous system for motor control, occurs high in the brain. During exercise, the exercise pressor reflex (EPR) provides sympathetic activation. During orthostasis, changes in the direction and magnitude of gravity cause sympathetic activation via the vestibular apparatus and activation of the circulatory centers [44].

The otoliths and semicircular canals of the inner ear are involved in eye movements and postural control, and blood pressure regulation during orthostasis is accomplished by feedforward control by these vestibular organs and feedback control by the baroreceptor reflex. Although blood pressure temporarily decreases during orthostasis, the vestibular organs, via the sympathetic nervous system, exert feedforward control, in which blood pressure begins to rise before it actually decreases. As a result, however, blood pressure is not maintained, but rather rises due to overregulation. This rise in blood pressure is corrected by feedback control through baroreceptor reflex, resulting in a slight increase in blood pressure in normal subjects. During orthostasis, the arterial blood pressure is thought to be regulated to minimize fluctuations in arterial blood pressure by the interaction between the vestibular arterial blood pressure reflex, which acts as a feedforward system, and the baroreceptor reflex, which acts as negative feedback [45]. It is possible that EAT regulates vestibular function by activating the baroreceptor reflex.

Serotonergic neurons in the nucleus accumbens and noradrenergic neurons in the LC receive vestibular input and are involved in vestibular autonomic reflexes. The histaminergic nervous system is involved in the development of vertigo symptoms. Vestibular input and the acetylcholine nervous system inhibit the noradrenergic nervous system and are involved in the development of vertigo symptoms. These nervous systems are thought to interact and influence vestibular function [46]. It is possible that EAT affects these nervous systems to regulate vestibular function.

Although orthostatic hypotension is often observed in the elderly, it has been reported that the arterial blood pressure control system is attenuated with age. It has also been reported that the decreased orthostatic tolerance seen in astronauts after their return to Earth is due to decreased function of the inner ear vestibular system [47]. It may be necessary to distinguish whether orthostatic dysfunction is due to impaired baroreceptor reflex function or impaired inner ear vestibular system function, It is possible that EAT regulates the vestibular arteriolar baroreflex by affecting the baroreceptor reflex and the inner ear vestibular system function by affecting the serotonergic and noradrenergic neuronal systems, histaminergic, acetylcholine neuronal system, and sympathetic nervous system.

EAT has been reported to be useful in suppressing vertigo symptoms in orthostatic dysregulation (OD), postural orthostatic tachycardia syndrome (POTS), myalgic encephalomyelitis/chronic fatigue syndrome (ME/CFS), etc. [3, 48]. It is thought that some of the vertigo symptoms that occur during positional changes may be due to blood pressure fluctuations caused by decreased sympathetic nerve activity. The suppression of parasympathetic activity by EAT stimulation oppositely stimulates sympathetic activity. Activation of baroreceptor reflex by EAT suppresses blood pressure variability and maintains and stabilizes blood pressure. Since EAT suppresses blood pressure variability during orthostasis, it may also influence the vestibular artery baroreceptor reflex. The sympathomimetic and blood pressure regulating effects of EAT may be effective in suppressing vertigo symptoms.

Effects of EAT on arrhythmias

In normal subjects, parasympathetic neurotransmission is dominant, and a decrease in cardiac parasympathetic tone or a change in the ratio of sympathetic to parasympathetic tone is a predictor for arrhythmia and death. Both parasympathetic and sympathetic nerves are involved in atrial fibrillation. Because of the abundance of acetylcholine-sensitive K channels in the atria, parasympathetic stimulation or acetylcholine administration shortens the refractory period of atrial muscle, and variations in the refractory period are responsible for the development of atrial fibrillation. Catecholamine administration also shortens the refractory period and causes atrial fibrillation. Group Ia antiarrhythmic agents with anticholinergic effects and group III agents with potassium channel blocking effects are effective in the treatment of parasympathetic-involved atrial fibrillation [49]. EAT may be effective in the treatment of parasympathetic-involved atrial fibrillation. Low-level vagus nerve stimulation has also been reported to prevent postoperative AF. One hour of auricular stimulation significantly shortened AF duration and reduced inflammatory cytokines. One hour of auricular stimulation per day over a six-month period has been reported to significantly reduce paroxysmal AF [50].

Therapeutic interventions for the maintenance of sinus rhythm (rhythm control) and heart rate regulation (rate control) also require a decision as to whether the treatment is to improve life expectancy or quality of life. Ablation and other therapies are available, but a decision must be made after weighing the risks and benefits. Cardiac plexus ablation (ganglionated plexi ablation), which intervenes in the endogenous autonomic nervous system, such as the autonomic plexus (ganglionated plexi: GP) located on the epicardial side of the left atrium, is an invasive treatment for the autonomic nervous system [51,52]. In contrast, EAT is considered a noninvasive to minimally invasive treatment that acts on the exogenous autonomic nervous system, including the brainstem, vagus nervous system, and sympathetic nervous system. EAT, which stimulates both sympathetic and parasympathetic nervous system activity and is expected to have a balancing effect, may have the potential to reduce the occurrence of atrial fibrillation.

Stress response induced by EAT

EAT affects sympathetic nervous system activity by acting as psychological and physical stress [53]. Psychological stress enhances central NA nervous system activity and stimulates sympathetic nervous system activity while simultaneously increasing arousal levels. The cell bodies of central NA-containing neurons (A1 to A7) are located from the medulla oblongata to the bridge. LC-NA neurons originating from A4 and A6 (locus ceruleus: LC) project to phylogenetically new areas such as the neocortex and hippocampus, where they are mainly associated with psychological stress [54]. Other non-LC-NA neurons originating from A1, A2, A3, A5, and A7 project to the hypothalamus, amygdaloid nucleus, and lateral nucleus of the spinal cord intermedius. It is directly connected to the centers of the sympatho-adrenal medullar (SAM) and hypothalamic-pituitary-adrenal (HPA) systems and is mainly related to physical stress [54]. Psychological stress is processed in the corticolimbic system and transmitted from the dorsomedial hypothalamus (DMH) to the paraventricular nucleus, which activates the HPA system by increasing corticotropin-releasing hormone (CRH) and through feedback control The LC and non-LC systems interact with each other [55].

The center of the defense response of the SAM system, known as Cannon's fight-or-flight emergency response, is located in the lateral part of the midbrain central gray matter, the periaqueductal gray (PAG) [56]. Somatic stress responses provide input to the lateral PAG and trigger either fight or flight responses. Stimuli such as visceral sensations or psychological stress can cause a freezing phenomenon in the ventral PAG, which is located at the intersection of ascending sensory information and input from higher centers, and different modes of input to the PAG can elicit different responses [57]. It is speculated that the initial stimulation by the EAT acts as a psychological stress and induces a freeze phenomenon, while the continuous stimulation by the EAT acts as a physical stress and induces a fight-or-flight response [53].

The HPA system suppresses the secretion of CRH and adrenocorticotropic hormone (ACTH) by negative feedback regulation to prevent the excessive transmission of stress stimuli. The HPA system is essential for adaptation to external environmental stimuli. However, persistent stressful stimuli can cause dysfunction in the HPA system and disrupt homeostasis, which maintains the constancy of the internal environment. Homeostasis assumes a state of static equilibrium, but that equilibrium is constantly changing. Rather than simply returning to the equilibrium point, the body maintains the internal environment by maintaining dynamic homeostasis through various reactions. Allostasis is a dynamic adaptive capacity based on predictive control, in which the internal environmental setpoint itself is dynamically adjusted to increase control efficiency. In other words, it maintains the stability of the internal environment by changing and adapting to acute stress [58]. EAT initially acts as an acute transient stress, but the body repeatedly adapts through repetitive EAT stimulation [53]. It is thought that EAT causes the SAM system to adapt so that it can respond quickly. When the allostatic response fails to function properly, the body's load accumulates and physical and mental modifications may occur, triggering an overreaction such as the Reilly phenomenon or a lack of adaptation [53].

Effect of EAT on trigeminal-parasympathetic reflexes

Nociceptive stimulation by chronic epipharyngitis stimulates polymodal receptors, which project to the hypothalamus via the trigeminopituitary tract. Ultimately, it is transmitted to the limbic system, which controls emotions and feelings. In contrast to primary pain (first pain) caused by high-threshold mechanical receptor stimulation, secondary pain (second pain) caused by polymodal receptor stimulation produces a hyperalgesic state and is remembered as more emotional pain (anxiety, discomfort, etc.) [59]. Stimulation of trigeminal visceral afferent fibers activates the trigeminal vascular pathway, which secretes neuropeptides such as SP, neurokinin A (NKA), and other tachykinins and CGRP, and induces neurogenic inflammation, including intracranial vasodilation, increased blood flow and vascular leakage. In addition, neurogenic inflammation is also induced from surrounding axonal side branch terminals via axonal reflexes. Neurogenic inflammation may create a vicious cycle that leads to a hyperalgesic state, prolonging symptoms such as headache, abnormal pharyngeal sensation, and chronic fatigue caused by chronic epipharyngitis [60].

Stimulation of trigeminal visceral afferent fibers also activates the trigeminocervical complex (TCC), which consists of the caudal subnucleus of the trigeminal spinal tract nucleus and the superior cervical medulla (C1 and C2) and projects to the superior salivary nucleus, where it synapses with postparasympathetic fibers in the pterygopalatine ganglion. The parasympathetic fibers then innervate the lacrimal gland and nasopharynx, triggering trigeminal and autonomic reflexes [30]. Chronic epipharyngitis may be a condition that is prone to triggering trigeminal and autonomic reflexes.

**Figure 3:**
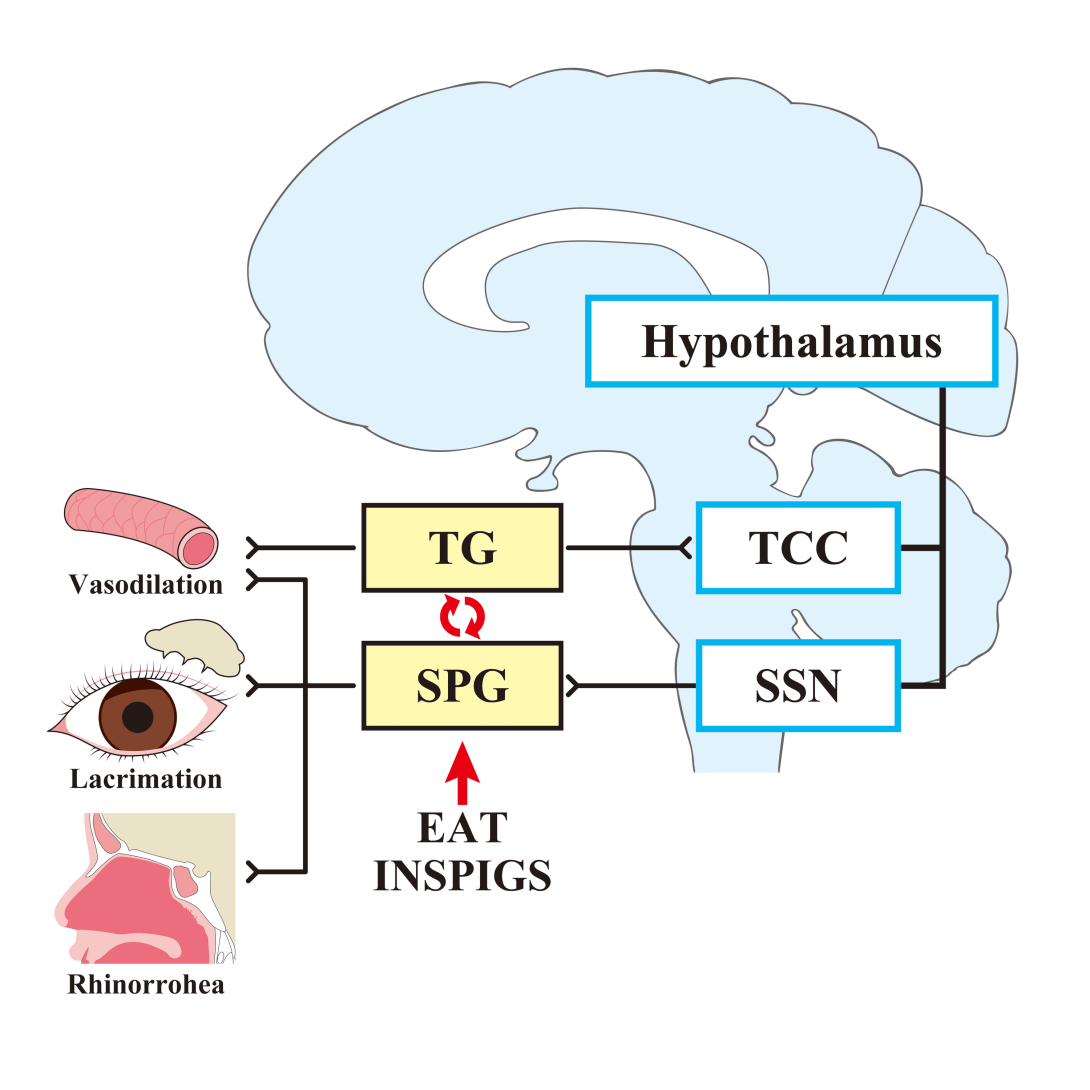
Effect of EAT on trigeminal and autonomic reflexes Stimulation of trigeminal visceral afferent fibers innervating cerebral and dural vessels activates the trigeminal vasculature, which in turn activates the thalamus and cortical structures involved in pain processing, resulting in pain. The hypothalamus is thought to act as a pain modulator. Stimulation of trigeminal visceral afferent fibers also projects to the superior salivary nucleus, synapses to postparasympathetic fibers via the pterygopalatine ganglion, and induces trigeminal and autonomic reflexes in the lacrimal gland and nasopharyngeal cavity. The trigeminovascular system and trigemino-autonomic reflexes influence each other and potentiate each other's effects through the release of vasoactive neuropeptides such as CGRP. EAT and INSPIGS may act on SPGs to suppressing trigeminal-parasympathetic reflexes and improve symptoms. SPG: superior salivary nucleus; SSN: superior salivary nucleus; TCC: trigeminocervical complex; TG: trigeminal ganglion; INSPIGS: intranasal sphenopalatine ganglion stimulation; EAT: epipharyngeal abrasive therapy

Although EAT directly acts on the epipharyngeal mucosa to improve chronic epipharyngitis, sympathetic stimulation by EAT activates the catecholamine receptor, the β2 adrenergic receptor (β2AR). β2AR stimulation inhibitoryly regulates the function of type 2 innate lymphoid cells (ILC2) and type 2 inflammatory responses [61]. CGRP promptly regulates the function of ILC2 [62], suggesting that EAT and CGRP stimulation act in an antagonistic manner. EAT may regulate the vasoreactivity of cerebral circulation to an appropriate level by suppressing neurogenic inflammation caused by CGRP and other factors. Parasympathetic stimulation by EAT may also activate cholinergic anti-inflammatory pathways that act on α7nAChR to suppress inflammatory cytokine production from macrophages and exert anti-inflammatory effects [63]. Since EAT has INSPIGS-like effects, it may be suppressing trigeminal-parasympathetic reflexes and improving symptoms, as well as interacting with immune cells to produce its effects. The effects of EAT on trigeminal and autonomic reflexes are shown in Figure 3.

Trigeminocardiac reflex (TCR) and vasovagal reflex (VVR)

The biological response to somatic nociceptive stimulation is often tachycardia and hypertension, whereas nociception of the trigeminal nerve region may result in bradycardia and hypotensive responses. Nociceptive stimulation reflexively increases blood pressure at the level of the spinal cord and brainstem, while pain input to the cerebral cortex acts on noradrenergic and serotonergic neurons of the descending pain suppression system to suppress sympathetic nerve activity and decrease blood pressure. Pain input to the trigeminal region inhibits sympathetic activity in the medullary trigeminospinal tract nucleus and lowers blood pressure. This is called the trigeminocardiac reflex (TCR [64]). The oculo-cardiac reflex (Aschner's reflex [65]), induced by strong pressure on the eyeball, is a stimulation of the first branch of the trigeminal nerve (oculocardial nerve) that suppresses sympathetic activity and stimulates parasympathetic activity, resulting in a decrease in blood pressure and bradycardia. TCR is defined as a decrease in blood pressure or heart rate of more than 20% from the reference value [66].

The TCR is a reflex produced by the transmission of a nociceptive stimulus applied to the trigeminal sensory branch to the parasympathetic nervous system, the vagus nerve, which induces parasympathetic arrhythmia, bradycardia, sinus arrest, sympathetic hypotension, apnea, or gastric hyperactivity. It is thought to have a similar physiological mechanism to the diving reflex [67], as it causes a regulation of systemic and cerebral circulation. Mental or physical stress may also suppress the sympathetic nervous system, inducing vasodilation, which in turn induces bradycardia due to vagal stimulation, resulting in the vasovagal reflex (VVR). The distinction between VVR and TCR is that VVR is preceded by an increase in sympathetic activity, whereas TCR is characterized by a sudden increase in parasympathetic activity [68].

EAT stimulation has both sympathomimetic and parasympathomimetic effects, and functions to maintain cerebral circulation by acting in a blood pressure-maintaining role on the heart and peripheral circulatory system and in a vasodilatory role on the cerebral circulatory system. Therefore, TCR and VVR are unlikely to be induced by EAT stimulation. However, since there is a time lag between the sympathetic and parasympathetic stimulating effects of EAT, it is possible that the antagonistic effects of EAT may be enhanced. It is also important to note that EAT stimulation may induce TCR and VVR because of differences in individual sensitivity to EAT stimulation [69].

Neuromodulation with EAT

Neuromodulation is a method of stimulating nerve tissue using devices such as deep brain stimulation (DBS) and spinal cord epidural stimulation (SCS) to interfere with the activity of the nerves. This treatment method uses electricity, magnetism, drugs, or other means to stimulate the nerves, thereby adjusting nerve function and relieving symptoms. The mechanisms of action range from directly interfering with nerve activity for immediate effect to modulating nerve excitability and inducing neural plasticity [70].

Vagus nerve stimulation (VNS) is used as a treatment for intractable epilepsy, depression, and migraine, and is thought to contribute to seizure suppression by modulating the activity of the nucleus accumbens and via noradrenergic nerves. It is also believed that VNS has an antiepileptic effect by broadly stabilizing the cerebral cortex via serotonergic and acetylcholine nerves. Stimulation of the NTS by VNS releases inhibitory transmitters such as GABA, decreases glutamine release from the caudal subnucleus of the trigeminal nucleus caudalis (TNC), and blocks pain perception, making VNS effective in treating intractable pain. VNS has also been reported to have effects on inflammatory diseases. VNS is thought to exert its anti-inflammatory effects mainly through three pathways: the hypothalamus-pituitary-adrenal (HPA) pathway, the cholinergic anti-inflammatory pathway (CAP), and the splenic anti-inflammatory pathway [38,71].

EAT has sympathomimetic and vagus nerve stimulating effects and is expected to have an intervention effect on the cardiovascular system and baroreceptor reflexes because of its ability to regulate autonomic nervous functions [72]. Various effects on the central and peripheral nervous systems may also be expected. EAT is a noninvasive treatment method and is expected to be a safe and inexpensive treatment without complications associated with surgery.

## Conclusions

The EAT has both sympathomimetic and parasympathomimetic effects and regulates autonomic functions. The EAT reflex is hierarchically integrated and reflexively regulates not only the pharyngeal reflex but also autonomic functions such as cerebral circulation, cardiovascular, endocrine gland, and stress response. EAT is expected to have a variety of effects on the central and peripheral nervous systems. EAT is a non-invasive therapy and is expected to be a safe and inexpensive treatment without complications associated with surgery.
